# A method for high-throughput functional imaging of single cells within heterogeneous cell preparations

**DOI:** 10.1038/srep39319

**Published:** 2016-12-16

**Authors:** Adam S. Neal, Austin M. Rountree, Jared R. Radtke, Jianzhu Yin, Michael W. Schwartz, Christiane S. Hampe, Jonathan D. Posner, Vincenzo Cirulli, Ian R. Sweet

**Affiliations:** 1 UW Diabetes Institute, Department of Medicine, University of Washington, Seattle, WA, 98195, USA; 2 Department of Mechanical Engineering, University of Washington, Seattle, WA 98195, USA; 3 Institute for Stem Cell and Regenerative Medicine, Department of Pharmacology, WA 98195, USA

## Abstract

Functional characterization of individual cells within heterogeneous tissue preparations is challenging. Here, we report the development of a versatile imaging method that assesses single cell responses of various endpoints in real time, while identifying the individual cell types. Endpoints that can be measured include (but are not limited to) ionic flux (calcium, sodium, potassium and hydrogen), metabolic responsiveness (NAD(P)H, mitochondrial membrane potential), and signal transduction (H_2_O_2_ and cAMP). Subsequent to fluorescent imaging, identification of cell types using immunohistochemistry allows for mapping of cell type to their respective functional real time responses. To validate the utility of this method, NAD(P)H responses to glucose of islet alpha versus beta cells generated from dispersed pancreatic islets, followed by the construction of frequency distributions characterizing the variability in the magnitude of each individual cell responses were compared. As expected, no overlap between the glucose response frequency distributions for beta cells versus alpha cells was observed, thereby establishing both the high degree of fidelity and low rate of both false-negatives and false-positives in this approach. This novel method has the ability not only to resolve single cell level functional differences between cell types, but also to characterize functional heterogeneity within a given cell type.

## A need for functional assessment of heterogeneous mixture of cells

A common challenge in cell biology is the need to assess the functional attributes of isolated primary cells in heterogeneous cell mixtures. One example involves studies of directed differentiation of stem cells toward a given cell type of interest. Differences in cell fate specification, inefficient transitions of a given cell phenotype through specific stages of development, and intrinsic heterogeneity existing within populations of progenitor cells^1^ can each result in complex admixtures of many distinct cell types, and identifying and characterizing individual cell types in that mixture can be challenging. Other examples include the need to identify and characterize cells isolated from primary tissues such as liver^2^,^3^, pancreatic islets^4^,^5^, brain^6^, cardiomyocytes^7^ or blood leukocytes^8^. Assessing cellular differences in drug toxicity within a given tissue preparation can also be confounded if, for example, a sparsely represented cell type, but not the major parenchymal cell type, is targeted and eliminated by the drug. The ability to discriminate between these selective drug effects requires high-throughput cellular analysis methods that are not currently available.

These examples highlight instances in which measures of bulk cell response are uninformative with respect to cell-specific behavior. Even homogeneous cell mixtures can be characterized by wide variability in individual cellular responses, the nature of which may be physiologically or pathophysiologically important to characterize^9^. Such challenges can be addressed through an approach to single cell functional assessment that permits statistical analysis of the distributions of the responses. Achieving this goal, however, requires either that the cells are purified prior to study or that steps are taken beforehand to enable specific cell types to be identified within a complex cell mixture.

## Limitations of current approaches

One approach to addressing these challenges is to sort and purify cells prior to study using Fluorescence Activated Cell Sorting (FACS)^10^, but this separation method can adversely affect cell function and viability. Specifically, fluid shear stress on cells during FACS separation can be both variable and much greater than occurs *in vivo*, causing variable impairment of both cell viability^11^,^12^ and cell functions such as gene expression^13^ and cell cycle^14^. Detection of the expression of genetic markers of adult pancreatic cell types typically involves cell fixation^15^. Furthermore, the binding of fluorescent antibodies to cell-specific surface markers that enables the separation process can inadvertently stimulate target cells, thereby affecting subsequent analysis. Finally, when the proportion of a given cell type in a cell mixture is low, both the yield and purity of separated cells can be low^16^, increasing the likelihood of contamination by unwanted cells. Alternative approaches include strategies to genetically tag cells of interest with a fluorescent marker that allows for simultaneous identification of the cell type while performing the real time imaging analysis^17^. Often, this is an effective solution, but the process of labeling cells may affect their function, takes a number of days before the probes are expressed and it is not always possible to efficiently label primary tissue.

## A novel systematic approach: Combining cell identification with single-cell, real-time analysis

Based on the above considerations, we sought to develop a method that *1)* identifies cell type after functional analysis (such that the identification procedure does not affect analysis of cell function), and *2)* enables a high throughput approach to cellular analysis such that functional data is obtained on sufficient numbers of rare cell types. In addition, we strove to produce a method that was simple to implement, relied on readily available imaging equipment, and could be carried out on tissue soon after harvesting so that impact of the method would be widespread. These goals were achieved through an approach in which cell location is preserved and mapped following functional analysis by patterning a micro-scale numeric grid on the bottom of the cell chamber. We then used immunohistochemical staining to link the response of individual cells to its cellular identity, thereby circumventing the need for their purification. To measure the response of a large number of cells in real time, such that frequency distributions can be generated and analyzed with high statistical resolution, we employed automated stage control of the fluorescent imaging microscope in the x-y plane.

Real-time fluorescent imaging endpoints that can be utilized by our method include but are not limited to measures of intracellular levels of ions (e.g., calcium^18^, sodium^18^, potassium, hydrogen^18^ and zinc), metabolic function (e.g., NAD(P)H^19^ and mitochondrial membrane potential), and signal transduction molecules such as cAMP^18^ or reactive oxygen species. To illustrate and validate the method, we endeavored to compare glucose-stimulated NAD(P)H responses, and also glucose-stimulated intracellular calcium response, by alpha vs. beta cells derived from dispersed pancreatic islets. Pancreatic alpha cells, which synthesize and secrete the hormone glucagon, constitute a relatively rare cell type comprising 15–20% of total islet cells^20^, whereas beta cells constitute a majority of islet cells. Alpha and beta cells have distinctly different functional responses to changes glucose, and how alpha cell mitochondria respond to changes in glucose is not well-established^21^,^22^,^23^, making it a good test case for evaluating the method. The fluorescent signal detected for NAD(P)H includes contributions from both NADH and NADPH, but the signal is dominated by mitochondrial NADH (which reflects the balance between NADH generated by glycolytic/TCA cycle and its oxidation in the electron transport chain)^24^. In this report we demonstrate the successful and reproducible functional phenotyping of rare cell types within a complex cell mixture using single cell imaging analysis on a platform that is amenable to automation and high throughput applications and will be readily usable by cell biologists. This approach, therefore, overcomes several limitations associated with conventional analysis of cell preparations isolated by FACS.

## Methods

### Chemicals and solutions

Perifusion analyses were performed using Krebs Ringer bicarbonate solution (KRB) containing 0.1% BSA as done previously^25^, except that 5 mM was substituted for 25 mM sodium bicarbonate. Glucose, streptozotocin, carbonyl cyanide 4-(trifluoromethoxy)phenylhydrazone (FCCP), and potassium cyanide (KCN) were all purchased from Sigma-Aldrich (St. Louis, MO). Culture media (RPMI-1640) was supplied by ThermoFisher Scientific (Waltham, MA), and heat-inactivated fetal bovine serum (FBS) was supplied by Atlanta Biologicals (Flowery Branch, GA).

### Isolation and culture of rat islets

Islets were harvested from Sprague-Dawley male rats weighing approximately 250 g (Charles River, Wilmington, MA) that were anesthetized by intraperitoneal injection of sodium pentobarbital (35 mg/230 g rat). Approval for all surgical procedures was obtained from the Institutional Animal Care and Use Committee at the University of Washington, and all experiments were performed according to the written regulations and guidelines. Preparation and purification of islets were carried out as previously described^26^,^27^. Islets were cultured overnight prior to the experiment in culture media supplemented with 10% fetal bovine serum at 37 °C.

### Cell culture

INS-1 832/13 cells (passage number 52 to 74) (henceforth referred to as INS-1 cells) were kindly provided by Dr. Christopher Newgard and were cultured in RPMI-1640 (Gibco, Grand Island, NY) supplemented with 2 mM L-Glutamine, 1 mM Pyruvate, 50 μM Beta-mercaptoethanol, 20 mM HEPES, heat-inactivated fetal bovine serum (10%) (Atlanta Biologicals, Lawrenceville, GA) and 1% penicillin/streptomycin as previously described^28^.

### Patterning the grid on the perifusion cover slip

**Figure 1:**
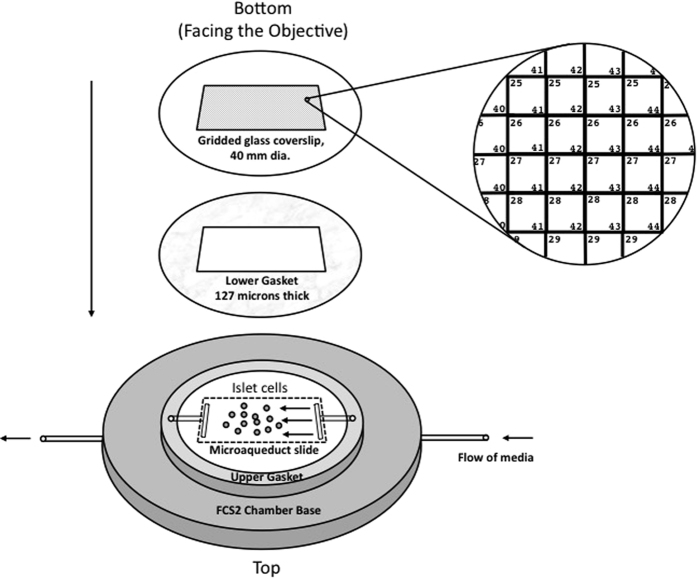
Schematic of the gridded fluidics chamber mapping cell positions. A Bioptechs FCS2, a closed system, parallel plate flow cell, was used per manufacturer’s instructions (see Methods section for details). Onto the bottom of the cover slip, a grid was patterned with chrome in order to generate a spatial map of the positions of the cells. The map of the cells was used to match the real time responses of the each cell to the identification of the cell type as reflected by subsequent immunostaining. Cells are not drawn to scale. Each square region in the grid was 150 microns × 150 microns, and the number in the upper left corner denoted the row, and the number in the lower right corner indicated the column.

Positioned as shown in Fig. 1, a grid of 17,689 squares measuring 150 microns per side was patterned onto one side of a 40 mm diameter perifusion coverslip supplied by Bioptechs (Butler, PA) in a microfabrication facility. Each square was dual-labeled with a combination of numbers and letters (25 × 15 microns) to give each square a unique identifier. The size of the box was set so that its perimeter was just inside the visible region of an image using a 40 × objective. The design of the coverslip was generated using CAD software (Layout Editor, juspertor UG), and a 5” AZ resist pre-coated Chrome mask was exposed using a laser writing tool (Heidelberg μPG101, Heidelberg Instruments, Germany), developed and subjected to Chrome etch to achieve the desired pattern. Coverslips were subsequently cleaned using isopropanol alcohol prior to the fabrication, spun coated with photoresist (AZ 1512, MicroChemicals, Germany) at 3000 *rpm* to yield a film thickness of 1 micron (6808P Spin Coater, Specialty Coating Systems, Indianapolis, IN), and soft-baked at 100 °C for 60 s before exposure to a broad wavelength aligner for 2 seconds with intensity of 21.6 mW/cm^2^ at 405 nm using the fabricated mask (ABM, Scotts Valley, CA). The coverslips were then developed and residues removed by a plasma ashing process (Trion Phantom Reactive Ion Etcher, Trion Technology, Tempe, AZ). The Chrome (approximately 50 *nm*) was evaporated onto the coverslip, (SEC-600, CHA Industries, Fremont, CA) and finally the lift-off process was performed in acetone by sonicating for 60 second.

### Dispersion of islets to single cells and plating them on perifusion cover slips

The day prior to their study, dispersed islet cells were prepared from isolated islets by gentle trituration of islets suspended in a phosphate buffered saline solution containing 0.125% trypsin/0.05 mM EDTA^29^. The islet suspension was triturated every minute (using a 1 mL pipet) until the islets were visibly dispersed (about 5–10 minutes), at which time the trypsin was deactivated by the addition of heat-inactivated fetal bovine serum. The dispersed islet cells were then seeded on to Bioptechs cover slips pre-coated with Matrigel (Becton Dickinson, Billerica, MA) on the reverse side of the etched grid, and allowed to attach for 1 h. Pre-coating of coverslips with Matrigel was done carried out 24 hours prior to use as follows. Matrigel was diluted 1:30 in PBS (containing no calcium or magnesium) and 200 μL were pipetted on to the cover slip. The cover slip was covered and allowed to set at 4 degrees C overnight. Single cells harvested from about 100 islets were seeded onto each coverslip, and incubated overnight in culture media supplemented with heat-inactivated fetal bovine serum (10%) at 37 °C.

### Fluidics system for maintaining cells in the fluorescence microscope

The perifusion chamber shown in Fig. 1 was used for maintaining and imaging plated islet cells. In place of the silicon lower gasket supplied by Bioptechs, gaskets were cut from Parafilm. A rectangular section was cut from a piece of Parafilm cut into a circle (d = 40 mm) so that the void created when the gasket was placed into the perifusion dish was fed and drained by the inlet and outlet ports. The system was readied for the experiment by first placing the upper gasket, the microaqueduct slide, and the lower gasket on to the FCS2 chamber base, which was then placed upside-down on the stage of a Nikon Eclipse Ti inverted microscope. This is depicted by a series of images included in Supplemental Information (Supplemental Fig. 1). The KRB solution (containing 5 mM sodium bicarbonate) was pumped through the inlet port, driven by a Masterflex L/S peristaltic pump (Model 7519-20, Cole Parmer, Vernon Hills, IL), for approximately 5 min until the void space in the lower gasket was filled. The pump was stopped, and the perifusion coverslip plated with islet cells was placed face down on to the lower gasket. The pieces thus assembled were secured in place with the FSC2 self-locking bracket. The assembly was then inverted so that the etched coverslip was contiguous with a 40 × oil immersion fluorescent objective, and placed within the stage opening. The pump was then turned on and set to a flow rate of 150 μL/min throughout the study, as described previously^30^.

### High throughput imaging of NAD(P)H and calcium

Fluorescence imaging of NAD(P)H in dispersed islet cells was performed with emission detected at 460 nm by a CoolSnap HQ2 CCD camera (Photometrics, Tucson, AZ) through a 40 × Super Fluor Nikon objective (DIC H/N2) during excitation at 360 nm via a Xenon lamp (Lambda LS-1620, Sutter Instrument Company, Novato, CA). NAD(P)H fluorescence integration time was 200 msec. The time sequence for data collection was thus, for a given region a fluorescence image was obtained, and the stage advanced to the next region where its fluorescence image was obtained and so on until all the regions specified were assessed. In this way, a fluorescence intensity was obtained for each cell for a given time point. This sequence was repeated for each time point, in this case every 120 seconds. With an acquisition time of 200 msec, the stage is capable of advancement to the next region at a maximal rate of ~3/second for a total of 180 regions/minute. With ~20 cells visible per region, the maximal rate of analysis is ~7200 cells/min in real time. The software package Elements^®^ (Nikon) was used to drive the data acquisition and movement of the stage controller. However, the program is unable to show data in real time while multiple positions are being assessed. To circumvent this problem, cell surface regions to be quantified were delineated manually for each visible region prior to real time analysis. Thus for each region scanned, Elements^®^ determined the fluorescence intensity for all delineated surface regions whether the cells were contained in that region or not. The data was exported to Excel (Microsoft) in real time where a customized “macro” binned the data for each actual cell residing in that window. At the completion of each protocol, the steady-state levels of relative fluorescence (RFU) during exposure of KCN and subsequently FCCP were measured and this data was used to normalize the RFU data. The normalization of the NAD(P)H signal was as a percent of RFU_FCCP_ and RFU_KCN_, defined as 0 and 100% respectively for each cell^31^. The imaging of calcium, after loading cells with the calcium-sensitive dye FURA-1, was carried out as previously described^32^.

### Immunohistochemical staining and imaging of cells on cover slip

Subsequent to real time imaging, the coverslip was removed from the flow chamber and submerged in 2% paraformaldehyde for 16 h. Immunohistochemical staining was carried out for insulin and glucagon using mouse anti-insulin monoclonal antibody CC9C10 (ATCC, Manassas, VA) and mouse anti-glucagon monoclonal antibody K79b810 (Abcam, Cambridge, UK). Antibody CC9C10 was directly labeled with Alexa Fluor 568 dye (excitation 578 nm and emission 603 nm) (Thermo Fisher Scientific, Waltham MA) and the glucagon antibody was visualized by use of goat anti-mouse IgG-FITC (excitation 494 nm and emission 522 nm) (Bio-Rad Laboratories, Hercules, CA). Cells were first analyzed for glucagon-positive cells, followed by staining for insulin-positive cells to avoid binding of the secondary antibody to the CC9C10 antibody using the same Nikon Eclipse Ti inverted microscope as used for real-time imaging.

### Data analysis

NAD(P)H responses were represented as the minimum percent changes of steady-state NAD(P)H in response to either increasing from 3 to 20 mM glucose, or a decrease from 20 to 3 mM glucose (calculated as the average of the NAD(P)H levels during the interval from 35–45 min after the change in glucose concentration). This approach was taken (rather than computing the average response) to prevent false positive findings that can result from signal drift. Frequency distributions were constructed by binning NAD(P)H responses in increments of 4% for each cell type. False positives were identified by glucagon-stained cells that responded like insulin-stained cells, and false negative were defined as insulin-stained cells that responded like glucagon-stained cells. Because calcium response to 20 mM glucose did not completely return to baseline after lowering glucose concentration back down to 4 mM, calcium responses were represented simply as the changes of steady-state fluorescence ratio in response to increasing from 4 to 20 mM glucose. Frequency distributions were constructed by binning calcium responses in increments of 0.1 for each cell type.

## Results

### Experimental overview

To determine whether metabolic responses of pancreatic alpha and beta cells can be distinguished from one another, studies were performed in dispersed islet cells. To determine the extent to which the alpha cell metabolic signature arises erroneously in a pure beta cell population (e.g., false positive rate), results from dispersed islet cells were compared to those obtained using an established beta cell line (INS-1 cells).

### Immunohistochemical staining of insulin and glucagon in dispersed cells

**Figure 2:**
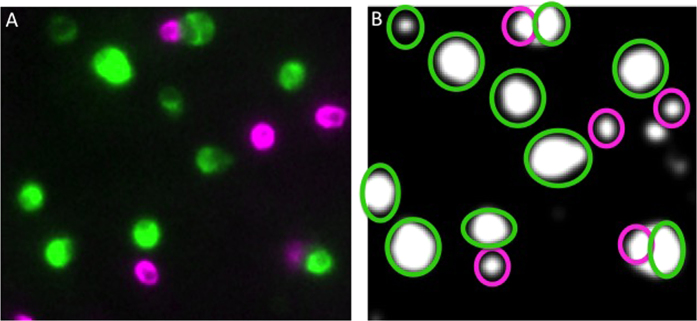
Mapping of fluorescent images of dispersed islet cells in a single grid region. Real time imaging of NAD(P)H was followed by subsequent immunostaining for cell type. (**A**) Islet cells immunostained for insulin (green) and glucagon (pink). (**B**) Autofluorescent NAD(P)H signal of islet cells at a single time point in the same grid. Pink circles delineate the cells identified as glucagon positive cells by immunostaining. Intensity was overexposed in order to clearly show the spatial position of the NAD(P)H signal for all cells relative to the immunostaining shown in (**A**).

Rat pancreatic islets were dispersed into single cells and seeded onto coverslips at a density of about 200,000 cells/plate. About 15–30 cells adhered to the Matrigel-coated plate per gridded region, where some cells situated in contact with other cells, while most made no direct contact with other cells; evidence of damage resulting from the isolation process was not observed. Bright-field exposures were taken prior to and after each fluorescent imaging experiment to count the number of cells that were not immunohistochemically stained. As expected, insulin and glucagon staining (carried out after the real time imaging analysis) was robust with virtually no overlap with respect to the identification of beta and alpha cells (Fig. 2A). In general, cells co-staining for both insulin and glucagon were not observed, although alpha and beta cells were occasionally aggregated into doublets. With ten regions imaged per time point, 224 cells were identified within those regions and immunohistochemically assessed for cell type over the course of three experiments. Of these cells, 183 were beta cells, 32 were alpha cells, and 12 did not stain for either hormone, possibly representing other islet cell types (e.g., those producing somatostatin, pancreatic polypeptide, or ghrelin).

### Real-time NAD(P)H responses of glucagon- and insulin-positive cells to glucose

NAD(P)H fluorescence was acquired from cells in each of the 10 regions. A fluorescent image was generated prior to the start of the real time experiment for each region and using the cursor, circles were drawn around the cells to be quantified by the software package Elements^®^. An example of an NAD(P)H autofluorescent image is shown for individual cells that were subsequently immunohistochemically identified (Fig. 2B), illustrating the mapping of NAD(P)H signal to cell type.

**Figure 3:**
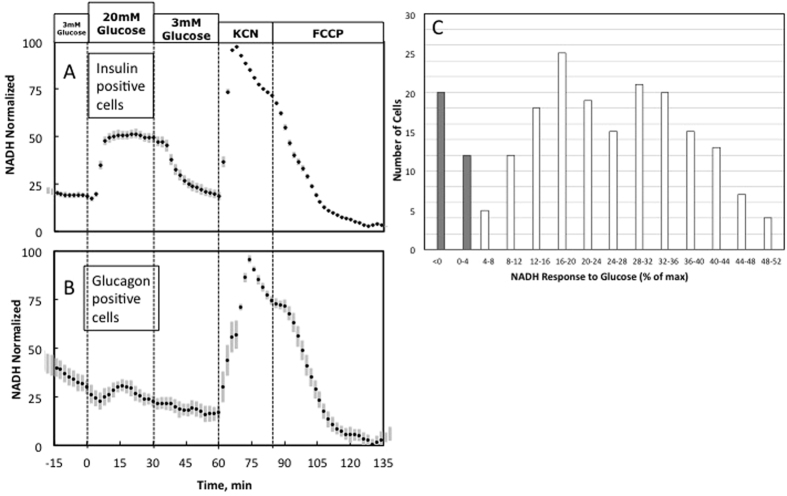
NAD(P)H responses to glucose by insulin-positive vs. glucagon-positive islet cells. (**A,B)** Kinetic data is the average of single cell responses to changes in glucose, KCN and FCCP by cells that were subsequently identified as (**A**) insulin- or (**B**) glucagon-positive (n = 183 and 32 respectively). KCN induced maximal NAD(P)H levels and the peak was set to 100%, and FCCP resulted in minimum levels and the trough was set to 0%. Results are the average of three separate experiments, where 12 cells were not stained. (**C**) Frequency distribution of NAD(P)H responses of islet cells to glucose for insulin- and glucagon-positive cells.

Among insulin-positive (beta) cells, an increase of ambient glucose concentration from 3 mM to 20 mM elicited a rapid and robust increase of NAD(P)H content that returned to baseline levels when glucose levels were reduced back to 3 mM (Fig. 3A). By comparison, the change of NAD(P)H content in glucagon-positive (alpha) cells to the same change of ambient glucose concentration was barely detectable (Fig. 3B). As a positive control, levels of NAD(P)H increased markedly in all cells during chemical hypoxia (cyanide), and they decreased in response to the mitochondrial uncoupler FCCP, confirming that the imaging methodology employed reliably detects changes of NAD(P)H content in glucagon- as well as insulin-positive cells. Following the real time imaging, immunohistochemical analysis was carried out as described above.

To establish the rate of false-positive and false-negative results for each cell type, a frequency distribution was constructed based on the minimum percent change of steady state NAD(P)H level in response to either an increase of ambient glucose concentration from 3 to 20 mM, or the subsequent decrease from 20 to 3 mM glucose. Analysis of cell number binned according to both cell type and NAD(P)H response in increments of 4% from 0–52% (Fig. 3C) demonstrated responses of 27 ± 0.9% *vs.* −5.0 ± 1.6% for beta *vs.* alpha cells, respectively. Only 5% of the cells were not definitively classified from the immunohistochemical staining, and these cells were not included in the analysis. There was virtually no overlap between the two cell types in the distribution of steady-state NAD(P)H responses to a change of glucose level.

### Real-time NAD(P)H responses to glucose in a pure population of insulin-positive cells

**Figure 4:**
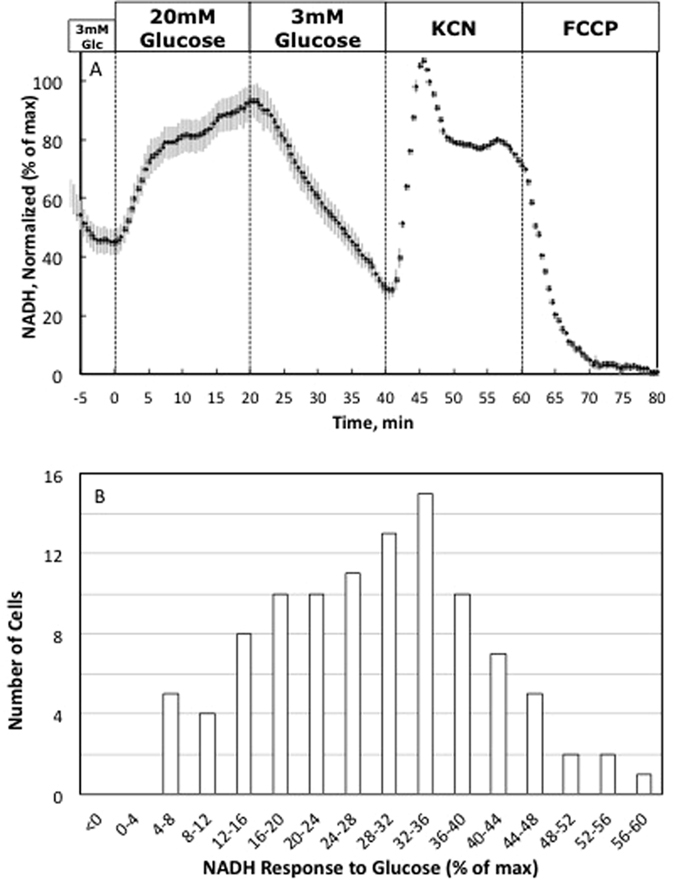
NAD(P)H responses to glucose by a pure population of insulin secreting cells (INS-1 832/13 cells). (**A**) Kinetic data is the average of single cell responses to changes in glucose, KCN and FCCP by all cells. Subsequently, all cells stained positive for insulin. Data was normalized as in Fig. 3. Results were generated in 1 experiment that analyzed 103 cells. (**B**) Frequency distribution of NAD(P)H responses of INS-1 cells to glucose. All INS-1 cells responded to glucose, and with a distribution similar to native beta cells.

To confirm the cell-type specificity of the NAD(P)H response to glucose, studies were repeated in a pancreatic beta cell line (INS-1 cells). In an analysis of 100 cells situated in 10 regions of the gridded coverslip, we observed NAD(P)H responses to changes of ambient glucose that resemble those of islet beta cells. Specifically, we observed a rapid increase of NAD(P)H content in response to an increased of ambient glucose levels from 3 mM to 20 mM that returned to baseline levels following the reduction of glucose concentrations back to 3 mM (Fig. 4A). There were however, differences in the kinetics of the two beta cell types, where the islet beta cells appeared to track the changes in glucose with much more fidelity. The frequency distribution of NAD(P)H changes in increments of 4% from 0–60% (composite response 28.5 ± 1.2%; Fig. 4B) was however comparable to that observed for dispersed islet beta cells, but not alpha cells (Fig. 3), and all cells stained positively for insulin, as expected. Our finding that no INS-1 cell responded with an increase of NAD(P)H of <4% establishes the robust specificity (i.e., low false positive rate) with which functionally distinct cells are detected by this method.

### Real-time intracellular calcium responses to glucose, followed by immunohistochemical staining of insulin and glucagon in dispersed cells

**Figure 5:**
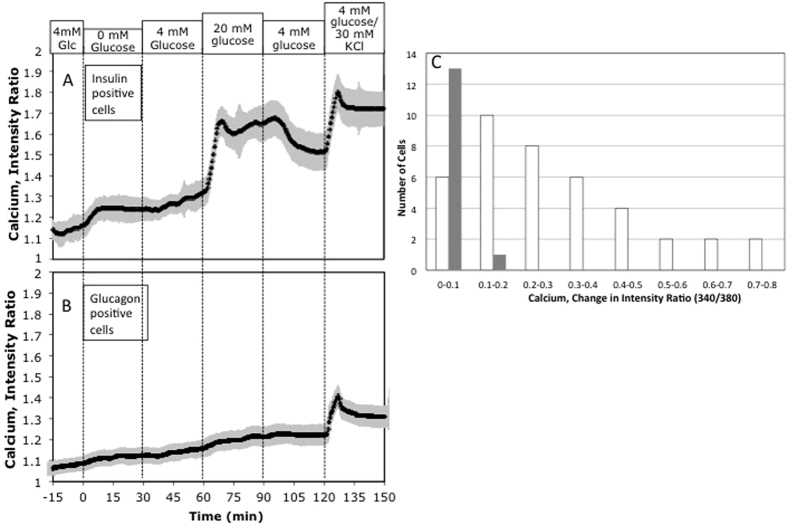
Intracellular calcium responses to glucose by insulin-positive vs. glucagon-positive islet cells. (**A,B**) Kinetic data is the average of single cell responses in intensity to changes in glucose and KCl concentrations by cells that were subsequently identified as (**A**) insulin- or (**B**) glucagon-positive (n = 40 and 14 respectively). Results are the average of two imaged regions in a single experiment containing approximately 27 cells per region. (**C**) Frequency distribution of calcium responses of islet cells to glucose for insulin- and glucagon-positive cells.

As with the NAD(P)H experiments on dispersed rat pancreatic islet cells, cells were immunohistochemically imaged for glucagon and insulin following the acquisition of the real time calcium data (data not shown). Two regions were imaged per time point, for a total of 54 islets cells assessed. Of these cells, 40 were beta cells, and 14 were alpha cells. Calcium fluorescence was acquired from cells in each of the 2 regions. Among insulin-positive (beta) cells, an increase of ambient glucose concentration from 4 mM to 20 mM elicited a rapid and robust increase of calcium content that partially returned to baseline levels when glucose levels were reduced back to 4 mM (Fig. 5A). By comparison, the change of calcium content in glucagon-positive (alpha) cells to the same change of ambient glucose concentration was barely detectable (Fig. 5B). A frequency distribution was constructed based on the percent change of steady state calcium level in response to an increase of ambient glucose concentration from 4 to 20 mM. Analysis of cell number binned according to both cell type and calcium response in increments of the change of fluorescence intensity ratio of 0.1 from 0–0.7 (Fig. 5C) demonstrated responses of 0.33 ± 0.054 *vs.* 0.051 ± 0.013 for beta *vs.* alpha cells, respectively. There was some, but not much overlap between the two cell types in the distribution of steady-state calcium responses to a change of glucose level. Thus, neither NAD(P)H nor calcium in alpha cells showed sensitivity to glucose changes in the physiologic range.

## Discussion

### Innovative features of a single-cell, live-imaging approach

The need to identify and functionally characterize unique cell types present within a heterogeneous cell mixture arises commonly across many areas of cell biology research. The standard approach to this challenge is to employ FACS to purify the cell type of interest prior to functional analysis. Limitations of this approach include the potentially deleterious impact of FACS on intrinsic cellular properties, such that responses of isolated cells to specific interventions may deviate from what might be observed had FACS not been used. This problem can be compounded by both cell loss and incomplete cellular purification, especially when the cell type(s) of interest constitute only a small proportion of the total number of cells. The alternative method described in this report allows cell-specific phenotyping in complex cell mixtures while circumventing the need for purification, provided that the cell type of interest (*1*) is amenable to immunohistochemical detection, and (*2*) displays functional responses that can be detected using a high-throughput imaging platform. To validate this alternative method, we studied mixtures of pancreatic alpha and beta cells derived from dispersed pancreatic islets. Our findings confirm that cell viability and function are preserved when studied using a flow culture system^33^. Indeed, the characteristic metabolic response to glucose was detected in virtually all INS-1 cells and pancreatic beta cells, and in no alpha cells establishing false negative and false positive rates of 0 in this assay. We also demonstrate that the assay system is amenable to high-throughput cell analysis, and can be applied to as few as a few hundred cells or up to thousands of cells.

We also report the use of frequency histograms to objectively quantify the analysis of cells in a complex mixture, since even cells of a single type cell type can exhibit a range of responses. Once the variability inherent in a particular functional read-out is established for each cell type, the sample size needed for accurate characterization can be determined. The method we used to achieve increased throughput was only partly automated (movement of the x-y plane stage controller was driven by Nikon’s Elements software). With further software development, delineating the cell regions where intensity is quantified will be automated, allowing thousands of cell responses to be analyzed in a single experiment.

### NAD(P)H and calcium response to glucose among alpha cells

Owing to the relatively small proportion of alpha cells in an islet, analysis of their response to glucose in dispersed islet cell preparations is difficult, especially since the predominant islet cell type (beta cells) is highly glucose-responsive. The single-cell, live-imaging approach introduced here offers a robust solution to this problem. The glucose-responsiveness of pancreatic beta cells depends in part on expression of glucokinase, a glucose-phosphorylating enzyme whose rate is very sensitive to changes in glucose in the physiological range (between 3 and 12 mM). In cells that express glucokinase, therefore, an increase of ambient glucose levels in this range is accompanied by increased oxidation of phosphorylated glucose, which increases cellular content of NAD(P)H^34^,^35^. Although some studies suggest that alpha cells contain glucokinase^36^,^37^, providing a mechanism for alpha cells to respond to glucose levels above 5 mM, other studies indicate that glucokinase in rat islets is exclusively localized to beta cells^38^. Moreover, some studies do not report reliable changes of alpha cells NAD(P)H content in response to an increase in glucose concentration^21^,^22^, another study reported a robust response^23^. The extent to which alpha cells exhibit increased glucose oxidation (and hence increased NAD(P)H content) in response to a physiological rise of ambient glucose levels, therefore, remains uncertain.

Using our live-cell microfluidics imaging system, we report that in stark contrast to what is observed in beta cells, alpha cells do not respond in a unidirectional manner to a physiological increase of ambient glucose concentration. The rate of alpha cell glucose metabolism, therefore, does not reliably vary in response to in physiological variation in ambient glucose concentrations, which may support a lack of a role for the K_ATP_ channel in alpha cell glucose sensing^21^. The glucose-insensitivity of alpha cells was further supported by intracellular calcium data that, consistent with previous studies on mouse islets^39^, also did not respond to changes in glucose. We acknowledge that the islet cell dispersion process may affect alpha cell function, but such an effect did not appear to affect glucose response of beta cells. Moreover, our finding that alpha cells displayed a robust increase (similar to that of beta cells) in NAD(P)H content in response to cyanide, and in calcium in response to KCl, suggests that all cells in our preparation were capable of displaying a robust metabolic and functional response.

### Beta cell NAD(P)H and calcium response to glucose

The NAD(P)H and calcium responses of dispersed beta cells to changes of ambient glucose that we observed were very similar to that displayed by intact islets^40^, including considerable variability in response magnitude. The latter is presumed to reflect the marked cell-to-cell heterogeneity in content of key proteins regulating beta cell metabolism, such that glucose-responsiveness of beta cells can vary from one cell to the next^9^,^41^,^42^,^43^. Although tissue dissociation into single cell preparations can affect cellular function, it is noteworthy that the method employed here effectively captures this functional heterogeneity. In addition, the dispersed cells responded to glucose more rapidly than the INS-1 cells, which continued to rise and fall 20 minutes after the change in glucose was instituted. As one might expect, the heterogeneity of the calcium responses in islet beta cells was somewhat higher than NAD(P)H heterogeneity, consistent with the notion that glucose-stimulated changes in metabolism are driving the changes in calcium.

### Applicability to other cell types and related considerations

The method presented here is optimally suited to the study of cell types that retain robust functional responses after isolation. As with other methods, the effects of dispersal and plating of cells should be considered in the analysis, both to determine the extent to which isolated cells behave differently than they do *in vivo* or in tissue explants^44^,^45^ and to determine optimal culture conditions. For example, available evidence suggests that the functionality of isolated islet cells improves when they are allowed either to adhere to an extracellular matrix^46^ or to re-aggregate into small cell clusters of as few as two or three cells^47^,^48^, whereas overly confluent cell preparations present problems of their own (e.g., reduced ability to spatially discriminate between cells present in multilayered compact cell clusters).

Another advantage of the system introduced here is that the fluidics systems we employed is designed to maintain flow rates and shear forces in a more physiological manner, which likely optimizes functional responsiveness of individual cells^49^, whether present as single cells or in small, adherent clusters of adjacent cells that may form monolayers. The microfluidic flow conditions also allow for constant replenishment of oxygen and nutrients, and concomitant removal of cell products, thereby reducing potential detrimental effects of paracrine cues that can accumulate in static systems^50^.

It is noteworthy that in our study, cell-cell interactions did not appear to alter the NAD(P)H response to glucose in either beta or alpha cells, as judged by the response of isolated cells *vs.* cells present in small or large clusters, suggesting that glucose metabolism of alpha and beta cells is not robustly affected by cell-cell interactions involving adhesion molecules. The method introduced here is positioned to be useful in assessing the impact of cell-cell interactions on discrete cellular functions, a possibility that awaits additional study. Possible examples include interactions between regulatory and effector T cells within different target tissues^51^, stem cells whose differentiation may be effected by contact with other cell types^52^, and, in general, in cell types that are functionally coupled through gap junctions^53^. These are all instances that offer an opportunity for further exploration and application of our mode of analysis in future studies.

### Summary

The technology we have developed that both identifies and characterizes individual cells, addresses a common problem in cellular biology involving characterization of cell-specific behavior within a heterogeneous mixture of cells. The approach rests on the micro-patterning of grids on the outer surface of imaging coverslips to allow for mapping of cell type to the cell’s respective functional characterization, and high throughput imaging via computer controlled movement of the microscope stage. We wish to emphasize that the methodology relies on imaging equipment that is widely available in most life science laboratories, and will be readily usable by cell biologists. Given the widespread need for quantifying both cell specific behavior and heterogeneity of function within the same cell type, and the wide range of endpoint imaging assays available, the approach should have applicability in many different fields of cell biology, physiology, medicine, and drug development.

## Additional Information

**How to cite this article:** Neal, S. A. *et al*. A method for high-throughput functional imaging of single cells within heterogeneous cell preparations. *Sci. Rep.* **6**, 39319; doi: 10.1038/srep39319 (2016).

**Publisher’s note:** Springer Nature remains neutral with regard to jurisdictional claims in published maps and institutional affiliations.

## Supplementary Material

Supplementary Information

## References

[b1] Hattori F. . Nongenetic method for purifying stem cell-derived cardiomyocytes. Nature methods 7, 61–66 (2010). 19946277 10.1038/nmeth.1403

[b2] Bartneck M. . Molecular response of liver sinusoidal endothelial cells on hydrogels. Mater Sci Eng C Mater Biol Appl 51, 64–72 (2015). 25842109 10.1016/j.msec.2015.02.045

[b3] Bartneck M. . Isolation and time lapse microscopy of highly pure hepatic stellate cells. Anal Cell Pathol (Amst) 2015, 417023 (2015). 26258009 10.1155/2015/417023 PMC4519541

[b4] Nielsen D. A., Lernmark A., Berelowitz M., Bloom G. D. & Steiner D. F. Sorting of pancreatic islet cell subpopulations by light scattering using a fluorescence-activated cell sorter. Diabetes 31, 299–306 (1982). 6130019 10.2337/diab.31.4.299

[b5] Nielsen O., Larsen J. K., Christensen I. J. & Lernmark A. Flow sorting of mouse pancreatic B cells by forward and orthogonal light scattering. Cytometry 3, 177–181 (1982). 6129116 10.1002/cyto.990030307

[b6] Daynac M. . Cell Sorting of Neural Stem and Progenitor Cells from the Adult Mouse Subventricular Zone and Live-imaging of their Cell Cycle Dynamics. J Vis Exp (2015). 10.3791/53247 PMC4692602 26436641

[b7] Banerjee I., Fuseler J. W., Price R. L., Borg T. K. & Baudino T. A. Determination of cell types and numbers during cardiac development in the neonatal and adult rat and mouse. Am J Physiol Heart Circ Physiol 293, H1883–1891 (2007). 17604329 10.1152/ajpheart.00514.2007

[b8] Ali R. A. . Nicotinic Acid Adenine Dinucleotide Phosphate Plays a Critical Role in Naive and Effector Murine T Cells but Not Natural Regulatory T Cells. J Biol Chem 291, 4503–4522 (2016). 26728458 10.1074/jbc.M115.681833 PMC4813477

[b9] Pipeleers D., Kiekens R., Ling Z., Wilikens A. & Schuit F. Physiologic relevance of heterogeneity in the pancreatic beta-cell population. Diabetologia 37 Suppl 2, S57–64 (1994). 7821741 10.1007/BF00400827

[b10] Van De Winkel M. & Pipeleers D. Autofluorescence-activated cell sorting of pancreatic islet cells: purification of insulin-containing B-cells according to glucose-induced changes in cellular redox state. Biochem Biophys Res Commun 114, 835–842 (1983). 6349638 10.1016/0006-291x(83)90857-4

[b11] Dimmeler S., Haendeler J., Rippmann V., Nehls M. & Zeiher A. M. Shear stress inhibits apoptosis of human endothelial cells. FEBS Lett 399, 71–74 (1996). 8980122 10.1016/s0014-5793(96)01289-6

[b12] Traub O. & Berk B. C. Laminar shear stress: mechanisms by which endothelial cells transduce an atheroprotective force. Arterioscler Thromb Vasc Biol 18, 677–685 (1998). 9598824 10.1161/01.atv.18.5.677

[b13] Toh Y. C. & Voldman J. Fluid shear stress primes mouse embryonic stem cells for differentiation in a self-renewing environment via heparan sulfate proteoglycans transduction. FASEB J 25, 1208–1217 (2011). 21183594 10.1096/fj.10-168971 PMC3058703

[b14] Davies P. F., Remuzzi A., Gordon E. J., Dewey C. F. Jr. & Gimbrone M. A. Jr. Turbulent fluid shear stress induces vascular endothelial cell turnover *in vitro*. Proc Natl Acad Sci USA 83, 2114–2117 (1986). 3457378 10.1073/pnas.83.7.2114 PMC323241

[b15] Dorrell C. . Isolation of mouse pancreatic alpha, beta, duct and acinar populations with cell surface markers. Mol Cell Endocrinol 339, 144–150 (2011). 21539888 10.1016/j.mce.2011.04.008 PMC3112273

[b16] Peng L. . Methodological limitations in determining astrocytic gene expression. Front Endocrinol (Lausanne) 4, 176 (2013). 24324456 10.3389/fendo.2013.00176 PMC3839565

[b17] Wile B. M., Ban K., Yoon Y. S. & Bao G. Molecular beacon-enabled purification of living cells by targeting cell type-specific mRNAs. Nat Protoc 9, 2411–2424 (2014). 25232937 10.1038/nprot.2014.154 PMC4326061

[b18] Tsien R. Y. Intracellular signal transduction in four dimensions: from molecular design to physiology. Am J Physiol 263, C723–728 (1992). 1329539 10.1152/ajpcell.1992.263.4.C723

[b19] Sweet I. R. . Endothelial inflammation induced by excess glucose is associated with cytosolic glucose 6-phosphate but not increased mitochondrial respiration. Diabetologia 52, 921–931 (2009). 19219423 10.1007/s00125-009-1272-4 PMC2741088

[b20] Michels J. E., Bauer G. E., Johnson D. & Dixit P. K. Morphometric analysis of the endocrine cell composition of rat pancreas following treatment with streptozotocin and nicotinamide. Exp Mol Pathol 44, 247–258 (1986). 3013674 10.1016/0014-4800(86)90039-0

[b21] Quoix N. . Glucose and pharmacological modulators of ATP-sensitive K^+^ channels control [Ca2^+^]c by different mechanisms in isolated mouse alpha-cells. Diabetes 58, 412–421 (2009). 19008345 10.2337/db07-1298 PMC2628615

[b22] Mercan D., Kadiata M. M. & Malaisse W. J. Differences in the time course of the metabolic response of B and non-B pancreatic islet cells to D-glucose and metabolized or non-metabolized hexose esters. Biochem Biophys Res Commun 262, 346–349 (1999). 10462477 10.1006/bbrc.1999.1219

[b23] Le Marchand S. J. & Piston D. W. Glucose suppression of glucagon secretion: metabolic and calcium responses from alpha-cells in intact mouse pancreatic islets. J Biol Chem 285, 14389–14398 (2010). 20231269 10.1074/jbc.M109.069195 PMC2863245

[b24] Patterson G. H., Knobel S. M., Arkhammar P., Thastrup O. & Piston D. W. Separation of the glucose-stimulated cytoplasmic and mitochondrial NAD(P)H responses in pancreatic islet beta cells. Proc Natl Acad Sci USA 97, 5203–5207 (2000). 10792038 10.1073/pnas.090098797 PMC25806

[b25] Sweet I. R. & Gilbert M. Contribution of calcium influx in mediating glucose-stimulated oxygen consumption in pancreatic islets. Diabetes 55, 3509–3519 (2006). 17130499 10.2337/db06-0400

[b26] Sweet I. R. . Regulation of ATP/ADP in pancreatic islets. Diabetes 53, 401–409 (2004). 14747291 10.2337/diabetes.53.2.401

[b27] Matsumoto S., Shibata S. & Kirchhof N. Immediate reversal of diabetes in primates following intraportal transplantation of porcine islets purified on a new histidine-lactoioniate-iodixanol gradient. Transplantation 67, S220 (1999).

[b28] Hohmeier H. E. . Isolation of INS-1-derived cell lines with robust ATP-sensitive K^+^ channel-dependent and -independent glucose-stimulated insulin secretion. Diabetes 49, 424–430 (2000). 10868964 10.2337/diabetes.49.3.424

[b29] Suckow A. T. . Transcellular neuroligin-2 interactions enhance insulin secretion and are integral to pancreatic beta cell function. J Biol Chem 287, 19816–19826 (2012). 22528485 10.1074/jbc.M111.280537 PMC3370167

[b30] Jung S. R., Reed B. J. & Sweet I. R. A highly energetic process couples calcium influx through L-type calcium channels to insulin secretion in pancreatic beta-cells. Am J Physiol Endocrinol Metab 297, E717–727 (2009). 19584201 10.1152/ajpendo.00282.2009 PMC2739700

[b31] Jung S. R., Reed B. J. & Sweet I. R. A highly energetic process couples calcium influx through L-type calcium channels to insulin secretion in pancreatic beta-cells. Am J Physiol Endocrinol Metab 297, E717–727 PMCID: PMC2739700 (2009). 19584201 10.1152/ajpendo.00282.2009 PMC2739700

[b32] Rountree A. M. . Control of insulin secretion by cytochrome c and calcium signaling in islets with impaired metabolism. J. Biol. Chem. 289, 19110–19119 (2014). 24841202 10.1074/jbc.M114.556050 PMC4081948

[b33] Sweet I. R. . Dynamic perifusion to maintain and assess isolated pancreatic islets. Diabetes Technol Ther 4, 67–76 (2002). 12017423 10.1089/15209150252924111

[b34] Sweet I. R., Li G., Najafi H., Berner D. & Matschinsky F. M. Effect of a glucokinase inhibitor on energy production and insulin release in pancreatic islets. Am J Physiol 271, E606–625 (1996). 8843758 10.1152/ajpendo.1996.271.3.E606

[b35] Matschinsky F. M. Banting Lecture 1995. A lesson in metabolic regulation inspired by the glucokinase glucose sensor paradigm. Diabetes 45, 223–241 (1996). 8549869 10.2337/diab.45.2.223

[b36] Heimberg H. . The glucose sensor protein glucokinase is expressed in glucagon-producing alpha-cells. Proc Natl Acad Sci USA 93, 7036–7041 (1996). 8692940 10.1073/pnas.93.14.7036 PMC38931

[b37] Bedoya F. J., Oberholtzer J. C. & Matschinsky F. M. Glucokinase in B-cell-depleted islets of Langerhans. J Histochem Cytochem 35, 1089–1093 (1987). 3305701 10.1177/35.10.3305701

[b38] Jetton T. L. & Magnuson M. A. Heterogeneous expression of glucokinase among pancreatic beta cells. Proc Natl Acad Sci USA 89, 2619–2623 (1992). 1557365 10.1073/pnas.89.7.2619 PMC48713

[b39] Rorsman P., Braun M. & Zhang Q. Regulation of calcium in pancreatic alpha- and beta-cells in health and disease. Cell Calcium 51, 300–308 (2012). 22177710 10.1016/j.ceca.2011.11.006 PMC3334273

[b40] Gilbert M., Jung S. R., Reed B. J. & Sweet I. R. Islet oxygen consumption and insulin secretion tightly coupled to calcium derived from L-type calcium channels but not from the endoplasmic reticulum. J Biol Chem 283, 24334–24342 (2008). 18593707 10.1074/jbc.M802097200 PMC2528984

[b41] Schuit F. C. Factors determining the glucose sensitivity and glucose responsiveness of pancreatic beta cells. Horm Res 46, 99–106 (1996). 8894663 10.1159/000185004

[b42] Giordano E., Bosco D., Cirulli V. & Meda P. Repeated glucose stimulation reveals distinct and lasting secretion patterns of individual rat pancreatic B cells. J Clin Invest 87, 2178–2185 (1991). 2040700 10.1172/JCI115251 PMC296977

[b43] Giordano E. . B-cell size influences glucose-stimulated insulin secretion. Am J Physiol 265, C358–364 (1993). 8368265 10.1152/ajpcell.1993.265.2.C358

[b44] Benninger R. K., Zhang M., Head W. S., Satin L. S. & Piston D. W. Gap junction coupling and calcium waves in the pancreatic islet. Biophys J 95, 5048–5061 (2008). 18805925 10.1529/biophysj.108.140863 PMC2586567

[b45] Benninger R. K. & Piston D. W. Cellular communication and heterogeneity in pancreatic islet insulin secretion dynamics. Trends Endocrinol Metab 25, 399–406 (2014). 24679927 10.1016/j.tem.2014.02.005 PMC4112137

[b46] Bosco D., Meda P., Halban P. A. & Rouiller D. G. Importance of cell-matrix interactions in rat islet beta-cell secretion *in vitro*: role of alpha6beta1 integrin. Diabetes 49, 233–243 (2000). 10868940 10.2337/diabetes.49.2.233

[b47] Bosco D., Orci L. & Meda P. Homologous but not heterologous contact increases the insulin secretion of individual pancreatic B-cells. Exp Cell Res 184, 72–80 (1989). 2676573 10.1016/0014-4827(89)90365-0

[b48] Parnaud G. . Cadherin engagement improves insulin secretion of single human beta-cells. Diabetes 64, 887–896 (2015). 25277393 10.2337/db14-0257

[b49] Shemesh J. . Flow-induced stress on adherent cells in microfluidic devices. Lab Chip 15, 4114–4127 (2015). 26334370 10.1039/c5lc00633c

[b50] Neal A. . Quantification of Low-Level Drug Effects Using Real-Time, *in vitro* Measurement of Oxygen Consumption Rate. Toxicological sciences: an official journal of the Society of Toxicology 148, 594–602 (2015). 26396153 10.1093/toxsci/kfv208 PMC4830255

[b51] Schmidt A., Oberle N. & Krammer P. H. Molecular mechanisms of treg-mediated T cell suppression. Front Immunol 3, 51 (2012). 22566933 10.3389/fimmu.2012.00051 PMC3341960

[b52] Moccia F., Tanzi F. & Munaron L. Endothelial remodelling and intracellular calcium machinery. Curr Mol Med 14, 457–480 (2014). 24236452 10.2174/1566524013666131118113410

[b53] Elias L. A. & Kriegstein A. R. Gap junctions: multifaceted regulators of embryonic cortical development. Trends Neurosci 31, 243–250 (2008). 18403031 10.1016/j.tins.2008.02.007 PMC2610634

