# Supplementary material for: A method for high-throughput functional imaging of single cells within heterogeneous cell preparations
Source: Sci Rep. 2016 Dec 16;6:39319. doi: 10.1038/srep39319 (PMC5159830; doi:10.1038/srep39319)
Supplement: Supplementary Information [file srep39319-s1.pdf]

Title

A method for high-throughput functional imaging of single cells within heterogeneous cell preparations

Authors and Affiliations

Adam Neal[1], Austin Rountree[1], Jared R. Radtke[1], Jianzhu Yin[2], Michael Schwartz[1], Christiane S. Hampe[1], Jonathan Posner[2], Vincenzo Cirulli[1,3], Ian Sweet[1§].

1. UW Diabetes Institute, Department of Medicine, University of Washington, Seattle, WA, 98195, U.S.A.
2. Department of Mechanical Engineering, University of Washington, Seattle, WA 98195, USA
3. Institute for Stem Cell and Regenerative Medicine, Department of Pharmacology, WA 98195, USA

§Corresponding Author: Ian Sweet
University of Washington
University of Washington Diabetes Institute
Seattle, WA 98108-1532
U.S.A.

Tel: 206 685-4775
Fax: 206 543-3567
E-mail: isweet@u.washington.edu

Supplemental FIGURE LEGEND

Supplemental Figure 1. Assembly of FCS2 perifusion chamber. A Bioptechs FCS2 was assembled per manufacturer's instructions (see Methods section and caption below each image for details).

Supplemental Figure 1A-D

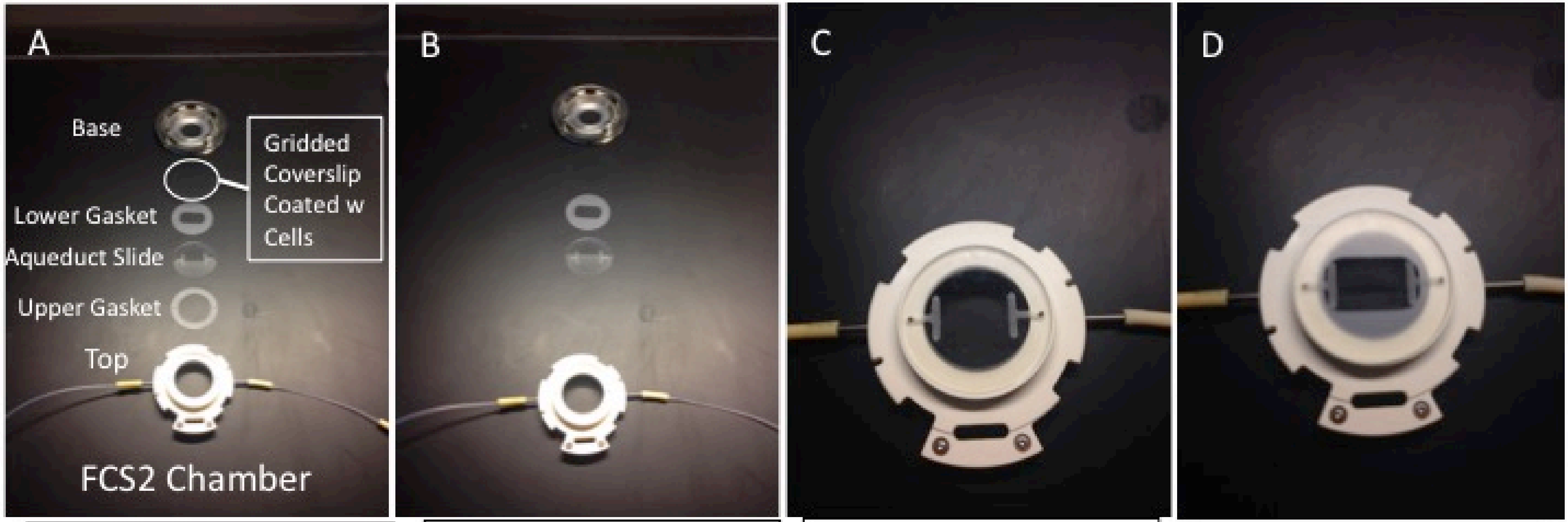

A: Assembling the Bioptechs FCS2 chamber. Perifusion chamber parts.

B: Step 1. Place Upper Gasket on to the Top of the chamber.

C: Step 2. Place Aqueduct Slide on the Upper Gasket.

D: Step 3. Place Lower Gasket on the Aqueduct Slide.

Supplemental Figure 1E-G

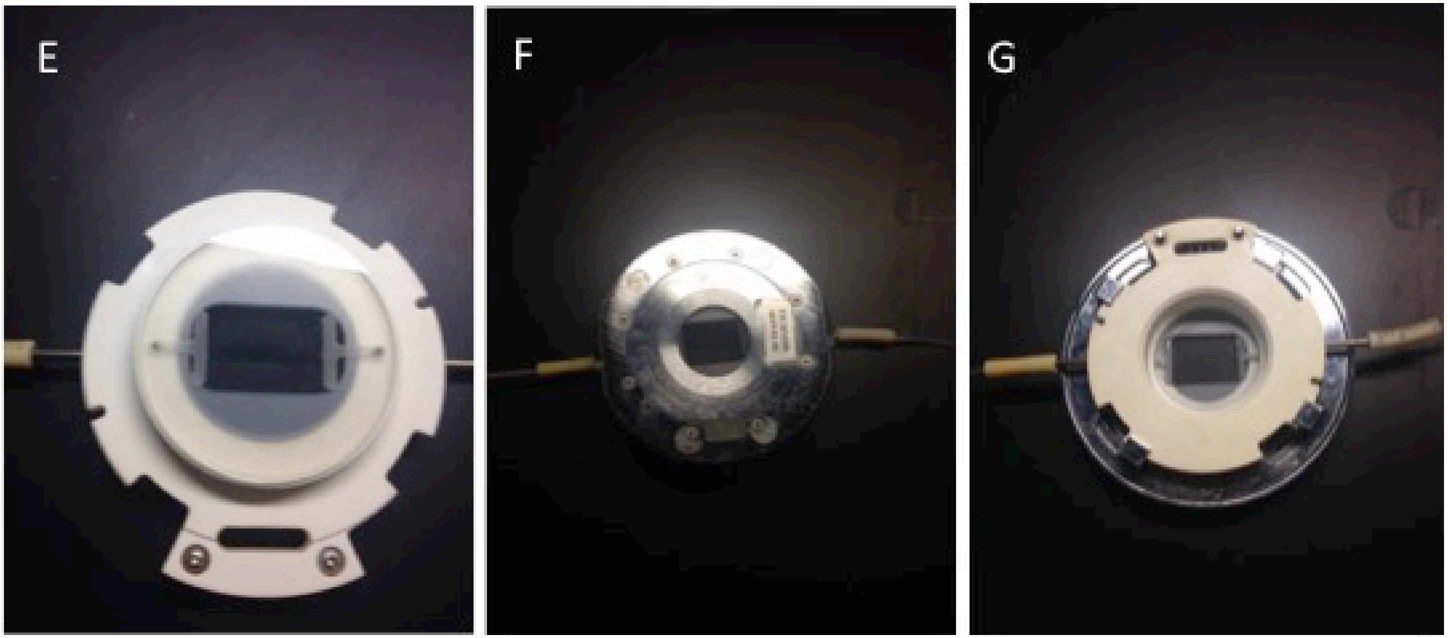

Step 4. Prior to placing the Gridded Cover Slip on to the Lower Gasket, perifusate is pumped in to the chamber until the liquid level is above the Lower Gasket. Then the Gridded Cover Slip is placed on to top of the Lower Gasket Cell Side down, being careful not to trap any bubbles.

Step 5. Once the Cover Slip is in place, and all of the layers are aligned, the Base is put on and locked in place.

Step 6. The whole device can now be turned over and then put in place in the microscope stage.
